# MicroRNA Changes Up to 24 h following Induced Hypoglycemia in Type 2 Diabetes

**DOI:** 10.3390/ijms232314696

**Published:** 2022-11-24

**Authors:** Manjunath Ramanjaneya, Ilham Bettahi, Krunal Pawar, Najeeb M. Halabi, Abu Saleh Md Moin, Thozhukat Sathyapalan, Abdul Badi Abou-Samra, Stephen L. Atkin, Alexandra E. Butler

**Affiliations:** 1 Qatar Metabolic Institute, Hamad Medical Corporation, Doha P.O. Box 3050, Qatar; 2 Translational Research Institute, Hamad Medical Corporation, Doha P.O. Box 3050, Qatar; 3 Amity Institute of Biotechnology, Amity University, Jaipur 201313, India; 4 Weill Cornell Medicine in Qatar, Education City, Qatar Foundation, Doha P.O. Box 3050, Qatar; 5 Royal College of Surgeons in Ireland Bahrain, Adliya 15503, Bahrain; 6 Academic Endocrinology, Diabetes and Metabolism, Hull York Medical School, Hull HU6 7RU, UK

**Keywords:** type 2 diabetes, hypoglycemia, miRNA, metabolic pathways

## Abstract

Hypoglycemia, as a complication of type 2 diabetes (T2D), causes increased morbidity and mortality but the physiological response underlying hypoglycemia has not been fully elucidated. Small noncoding microRNA (miRNA) have multiple downstream biological effects. This pilot exploratory study was undertaken to determine if induced miRNA changes would persist and contribute to effects seen 24 h post-hypoglycemia. A parallel, prospective study design was employed, involving T2D (n = 23) and control (n = 23) subjects. The subjects underwent insulin-induced hypoglycemia (2 mmol/L; 36 mg/dL); blood samples were drawn at baseline, upon the induction of hypoglycemia, and 4 h and 24 h post-hypoglycemia, with a quantitative polymerase chain reaction analysis of miRNA undertaken. The baseline miRNAs did not differ. In the controls, 15 miRNAs were downregulated and one was upregulated (FDR < 0.05) from the induction of hypoglycemia to 4 h later while, in T2D, only four miRNAs were altered (downregulated), and these were common to both cohorts (miR-191-5p; miR-143-3p; let-7b-5p; let-7g-5p), correlated with elevated glucagon levels, and all were associated with energy balance. From the induction of hypoglycemia to 24 h, 14 miRNAs were downregulated and 5 were upregulated (FDR < 0.05) in the controls; 7 miRNAs were downregulated and 7 upregulated (FDR < 0.05) in T2D; a total of 6 miRNAs were common between cohorts, 5 were downregulated (miR-93-5p, let-7b-5p, miR-191-5p, miR-185-5p, and miR-652-3p), and 1 was upregulated (miR-369-3p). An ingenuity pathway analysis indicated that many of the altered miRNAs were associated with metabolic and coagulation pathways; however, of the inflammatory proteins expressed, only miR-143-3p at 24 h correlated positively with tumor necrosis factor-α (TNFa; *p* < 0.05 and r = 0.46) and negatively with toll-like receptor-4 (TLR4; *p* < 0.05 and r = 0.43). The MiRNA levels altered by hypoglycemia reflected changes in counter-regulatory glucagon and differed between cohorts, and their expression at 24 h suggests miRNAs may potentiate and prolong the physiological response. Trial registration: ClinicalTrials.gov NCT03102801.

## 1. Introduction

Severe hypoglycemia has been associated with severe adverse cardiovascular events (MACE) and mortality in landmark trials of diabetes (for example, ACCORD, ADVANCE, ORIGIN, and DEVOTE) [1,2,3,4]; this suggests that a higher frequency of hypoglycemic events is directly linked with the poor outcomes. Endothelial and thrombotic dysfunction, which are strongly linked to diabetes complications, have been associated with hypoglycemia [5,6,7]. Their underlying mechanisms are known to include oxidative stress [8], increased inflammation [9], and the heat-shock protein response [10], but many of the fundamental underlying mechanistic dysfunctions that result from hypoglycemia are still unclear.

MicroRNAs (miRNAs) are non-coding RNAs that regulate gene expression post-transcriptionally and are approximately 22 nucleotides in length; their inhibitory actions result in mRNA cleavage, translational repression, and mRNA decay [11,12,13,14]. Each miRNA sequence may attenuate multiple target mRNA expressions, thus impacting many different biological and cellular pathways. MiRNAs have been inculpated in a variety of disease states, encompassing obesity, diabetes, and their related co-morbidities [15]. A growing body of the literature has reported stress-induced miRNA deregulation at the transcriptional level as well as at the processing, subcellular localization, and functioning levels [16]; further, accumulating evidence has confirmed the pivotal role that non-coding RNAs, notably miRNAs, play in the stress responses of cells [17]. For example, within diabetes, miR-126 participates in vascular repair [18], miR-146 is involved with oxidative stress and proinflammatory factors [19], the knockdown of miR-185 may provoke oxidative stress [20,21], and miR-503 inhibition restores endothelial dysfunction [22]. In a recent publication from our group relating to the same clinical study performed on the same patients but reporting only changes in miRNA from baseline to hypoglycemia, no changes in miRNA that passed the false detection rate were seen at the point of hypoglycemia, though the top altered miRNAs were associated with neurological disease and organismal abnormalities [23]. In a small hypoglycemia study restricted to 14 patients with type 2 diabetes, a selective miRNA analysis (chosen based upon platelet expression, namely, hsa-miR-129-2-3p, hsa-miR-223-5p, hsa-miR-223-3p, hsa-miR-16-5p, hsa.miR-15b-5p, hsa-miR-15b-3p, hsa-miR-15a-5p, hsa-miR-126-5p, hsa-miR-126-3p, and hsa-miR-106a.5p) was performed; the results at 1- and 7-days following the hypoglycemic event revealed increased levels of miR-126, miR-223, miR-16-5p, miR-15a, miR-15b, and miR-106a-5p [24].

Therefore, based on our previous results and those of others, this pilot exploratory study was undertaken to explore the hypothesis that hypoglycemia would induce miRNA changes that would occur following hypoglycemia with their persistence up to 24 h after the event.

## 2. Results

The characteristics (demographic and clinical) of the study subjects are detailed in Table 1. Those with T2D had short disease duration, were more obese, and had lower total cholesterol and HDL-cholesterol versus controls.

### 2.1. Baseline Comparison

At baseline, no differences in the miRNAs between T2D and the controls were detected.

### 2.2. Acute Changes in Response to a Hypoglycemia Insult: Changes in Controls and T2D from Hypoglycemia to 4 h (Table 2)

In T2D, only four miRNAs were altered (downregulated) (FDR < 0.05) from the induction of hypoglycemia to 4 h and these four—miR-191-5p, miR-143-3p, let-7b-5p, and let-7g-5p—were common to both T2D and the control cohorts (Table 2).

**Table 2:** MiRNAs that changed from the point of hypoglycemia to 4 h post-hypoglycemia in control and type 2 diabetes (T2D) subjects in the validation experiment. Significant changes in miRNAs are shown by fold change (Rq). It is evident that more miRNA changes (n = 16) were seen in control subjects than in subjects with T2D (n = 4), with all but one miRNA being downregulated. The miRNAs highlighted in blue were common to both control and T2D, and it is notable that all four of the altered miRNAs in T2D were also altered in control subjects. FDR, false discovery rate. Rq, relative level of miRNA expression.

Control Subjects (n = 23)				T2D Subjects (n = 23)			
Hypoglycemia to 4 h After			n = 16	Hypoglycemia to 4 h After			n = 4
Target Name	Rq (Fold Change)	FDR	Regulation	Target Name	Rq (Fold Change)	FDR	Regulation
hsa-miR-338-3p_478037_mir	3.0	0.001	Down	hsa-miR-191-5p_477952_mir	2.5	0	Down
hsa-miR-223-3p_477983_mir	2.9	0.001	Down	hsa-let-7b-5p_478576_mir	3.9	0.001	Down
hsa-miR-191-5p_477952_mir	2.5	0	Down	hsa-miR-143-3p_477912_mir	2.2	0.009	Down
hsa-miR-143-3p_477912_mir	2.4	0.001	Down	hsa-let-7g-5p_478580_mir	1.7	0.038	Down
hsa-let-7b-5p_478576_mir	2.2	0.002	Down				
hsa-miR-324-5p_478024_mir	2.1	0.007	Down				
hsa-miR-652-3p_478189_mir	2.0	0.02	Down				
hsa-miR-181b-5p_478583_mir	1.9	0.041	Down				
hsa-miR-186-5p_477940_mir	1.9	0.001	Down				
hsa-miR-24-3p_477992_mir	1.8	0.04	Down				
hsa-miR-26a-5p_477995_mir	1.8	0.001	Down				
hsa-miR-181a-5p_477857_mir	1.8	0.044	Down				
hsa-miR-339-5p_478040_mir	1.8	0.004	Down				
hsa-let-7g-5p_478580_mir	1.7	0	Down				
hsa-miR-126-3p_477887_mir	1.5	0.042	Down				
hsa-miR-195-5p_477957_mir	1.638	0.027	Up				

In the controls, a total of 16 miRNAs changed (FDR < 0.05) from hypoglycemia to 4 h after its induction; 15 were downregulated and one was upregulated, as shown in Table 2. Those twelve altered miRNAs from the induction of hypoglycemia to 4 h later that were specific to the controls were miR-338-3p, miR-223-3p, miR-324-5p, miR-652-3p, miR-181b-5p, miR-186-5p, miR-24-3p, miR-26a-5p, miR-181a-5p, miR-339-5p, miR-126-3p, and miR-195-5p.

### 2.3. Extended Effects of a Hypoglycemia Insult: Changes in Controls and T2D from Hypoglycemia to 24 h (Table 3)

In the controls, a total of 19 miRNAs changed (FDR < 0.05) from the hypoglycemia insult to 24 h later; 14 were downregulated and 5 were upregulated, as shown in Table 3. Of those 19, miRNAs, the 13 altered miRNAs specific only to the controls were miR-17-5p, miR-143-3p, miR-20a-5p, miR-223-3p, miR-186-5p, miR-338-3p, let-7g-5p, miR-324-5p, miR-339-5p, miR-10a-5p, miR-505-3p, miR-99b-5p, and miR-885-5p.

**Table 3:** MiRNAs that changed from the point of hypoglycemia to 24 h post-hypoglycemia in control (n = 23) and type 2 diabetes (T2D, n = 23) subjects in the validation experiment. The changes in miRNA from the point of hypoglycemia to 24 h post-hypoglycemia closely reflect the changes from baseline to 24 h post-hypoglycemia, as shown in Supplementary Table S1B. Here, the numbers of altered miRNAs were similar between controls (n = 19) and T2D (n = 14) subjects. In controls, the majority of miRNAs were downregulated (n = 14) and 5 were upregulated. In T2D, half were downregulated (n = 7) and half upregulated (n = 7). Those miRNAs highlighted in blue were common to both control and T2D groups. In accordance with the changes in miRNA from baseline to 24 h post-hypoglycemia (Supplementary Table S1B), 6 miRNAs that had altered from hypoglycemia to 24 h post-hypoglycemia were common to both groups, of which 5 were downregulated and only 1 was upregulated. FDR, false discovery rate; Rq, relative level of miRNA expression.

Control Subjects (n = 23)				T2D Subjects (n = 23)			
Hypoglycemia to 24 h			n = 19	Hypoglycemia to 24 h			n = 14
Target Name	Rq (Fold Change)	FDR	Regulation	Target Name	Rq (Fold Change)	FDR	Regulation
hsa-miR-17-5p_478447_mir	2.8	0.001	Down	hsa-miR-484_478308_mir	3.6	0.03	Down
hsa-miR-93-5p_478210_mir	2.6	0.001	Down	hsa-miR-652-3p_478189_mir	3.2	0.007	Down
hsa-let-7b-5p_478576_mir	2.5	0	Down	hsa-miR-191-5p_477952_mir	2.8	0.003	Down
hsa-miR-191-5p_477952_mir	2.5	0	Down	hsa-miR-93-5p_478210_mir	2.5	0.027	Down
hsa-miR-143-3p_477912_mir	2.4	0	Down	hsa-miR-106b-5p_478412_mir	2.3	0.016	Down
hsa-miR-20a-5p_478586_mir	2.2	0.001	Down	hsa-let-7b-5p_478576_mir	2.3	0.007	Down
hsa-miR-223-3p_477983_mir	2.2	0.043	Down	hsa-miR-185-5p_477939_mir	1.9	0.01	Down
hsa-miR-186-5p_477940_mir	2.2	0	Down	hsa-miR-21-5p_477975_mir	1.6	0.002	Up
hsa-miR-338-3p_478037_mir	2.1	0.031	Down	hsa-miR-29b-3p_478369_mir	1.7	0.015	Up
hsa-miR-185-5p_477939_mir	2.0	0.011	Down	hsa-miR-424-5p_478092_mir	1.7	0.007	Up
hsa-miR-652-3p_478189_mir	2.0	0.001	Down	hsa-miR-126-5p_477888_mir	1.8	0.002	Up
hsa-let-7g-5p_478580_mir	1.9	0.004	Down	hsa-miR-146a-5p_478399_mir	1.8	0.019	Up
hsa-miR-324-5p_478024_mir	1.7	0.01	Down	hsa-miR-369-3p_478067_mir	2.7	0.009	Up
hsa-miR-339-5p_478040_mir	1.5	0.021	Down	hsa-miR-365a-3p_478065_mir	6.7	0.019	Up
hsa-miR-10a-5p_479241_mir	1.6	0.019	Up				
hsa-miR-505-3p_478145_mir	1.9	0.001	Up				
hsa-miR-99b-5p_478343_mir	2.2	0.042	Up				
hsa-miR-369-3p_478067_mir	2.3	0.031	Up				
hsa-miR-885-5p_478207_mir	3.2	0.004	Up				

In T2D, a total of fourteen miRNAs changed (FDR < 0.05) from the induction of hypoglycemia to 24 h post-hypoglycemia; seven were downregulated and seven were upregulated, as shown in Table 3. Of those 14 miRNAs, 8 altered miRNAs were specific only to T2D: miR-484, miR-106b-5p, miR-21-5p, miR-29b-3p, miR-424-5p, miR-126-5p, miR-146a-5p, and miR-365a-3p.

Six altered miRNAs were common between the controls and T2D: miR-93-5p, let-7b-5p, miR-191-5p, miR-185-5p, and miR-652-3p were downregulated and only miR-369-3p was upregulated.

### 2.4. Changes from Hypoglycemia Common to Both the 4 h and 24 h Timepoints in Controls (Table 2 and Table 3)

In the controls, circulating levels of 10 miRNAs were altered—all downregulated—from the induction of hypoglycemia at both the 4 h and 24 h timepoints: miR-338-3p, miR-223-3p, miR-191-5p, miR-143-3p, let-7b-5p, miR-324-5p, miR-652-3p, miR-186-5p, miR-339-5p, and let-7g-5p.

The circulating levels of six miRNAs were altered at 4 h but had reverted to baseline by 24 h post-hypoglycemia in the controls: miR-181b-5p, miR-24-3p, miR-26a-5p, miR-181a-5p, miR-126-3p, and miR-195-5p.

The circulating levels of nine miRNAs were not altered at 4 h but were at the 24 h time point in the controls: miR-17-5p, miR-93-5p, miR-20a-5p, miR-185-5p, miR-10a-5p, miR-505-3p, miR-99b-5p, miR-369-3p, and miR-885-5p.

### 2.5. Changes from Hypoglycemia Common at Both the 4 h and 24 h Timepoints in T2D (Table 2 and Table 3)

In T2D, the circulating levels of two miRNAs were altered, both of which were downregulated, from the induction of hypoglycemia at both the 4 h and 24 h timepoints: miR-191-5p and let-7b-5p.

The circulating levels of two miRNAs were altered from the induction of hypoglycemia at 4 h but had reverted to baseline by 24 h post-hypoglycemia: miR-143-3p and let-7g-5p.

The circulating levels of twelve miRNAs were not altered from the hypoglycemia insult at 4 h but were at the 24 h time point in T2D: miR-484, miR-652-3p, miR-93-5p, miR-106b-5p, miR-185-5p, miR-21-5p, miR-29b-3p, miR-424-5p, miR-126-5p, miR-146a-5p, miR-369-3p, and miR-365a-3p.

### 2.6. Changes from Baseline Common at Both the 4 h and 24 h Timepoints in Controls (Supplementary Table S1)

In the controls, the circulating levels of nine miRNAs were altered, all downregulated, from baseline at both the 4 h and 24 h timepoints: miR-17-5p, let-7b-5p, miR-191-5p, miR-652-3p, miR-186-5p, let-7g-5p, miR-143-3p, miR-324-5p, and miR-151a-3p.

The circulating levels of four miRNAs were altered from baseline at 4 h but had reverted to baseline at 24 h post-hypoglycemia in the controls: miR-223-3p, miR-338-3p, miR-26a-5p, and miR-339-5p.

The circulating levels of seven miRNAs were not altered from baseline at 4 h but were at the 24 h time point: miR-93-5p, miR-20a-5p, miR-185-5p, miR-125b-5p, miR-505-3p, miR-369-3p, and miR-885-5p.

### 2.7. Changes from Baseline Common at Both the 4 h and 24 h Timepoints in T2D (Supplementary Table S1)

In T2D, the circulating levels of four miRNAs were altered, all downregulated, from baseline both at the 4 h and 24 h timepoints: miR-191-5p, let-7b-5p, miR-143-3p, and miR-365a-3p.

The circulating levels of eleven miRNAs were not altered from baseline at 4 h but were at the 24 h time point: miR-484, miR-652-3p, miR-93-5p, miR-106b-5p, miR-185-5p, miR-424-5p, miR-146a-5p, miR-126-5p, miR-21-5p, miR-10a-5p, and miR-369-3p.

### 2.8. Correlation Analyses

Correlation analyses were performed on the four miRNAs that were downregulated from the induction of hypoglycemia to 4 h later (miR-191-5p, miR-143-3p, let-7b-5p, and let-7g-5p) that were common to both T2D and control cohorts. Correlations were performed with selected proteins of interest based on the published literature at the 4 and 24 h post-hypoglycemia timepoints [6]. Accordingly, let-7g-5p was correlated with kallistatin; miR-191-5p was correlated with platelet factor 4 (PF4), P-selectin, platelet glycoprotein VI, plasminogen activator inhibitor-1 (PAI-1), Heparin cofactor 2, plasmin, plasminogen, alpha-2-antiplasmin, D-dimer, coagulation factors X and XI, and von Willebrand factor (vWF); miR-143-3p was correlated with interleukin 6 and 10 (IL6, IL10), interferon gamma (IFNg), tumor necrosis factor alpha (TNFa), and toll-like receptor 4 (TLR4); and let-7b-5p was correlated with IL6, IL10, IFNg, and TLR4.

Only in the T2D cohort were there significant correlations, and only with miR-143-3p at 24 h that correlated positively with TNFa (*p* < 0.05, r = 0.46) and negatively with TLR4 (*p* < 0.05, r = 0.43).

Glucagon, a major component of the counter-regulatory response, demonstrated an appropriate response in both the T2D and control cohorts following the hypoglycemia insult (Supplementary Figure S1). Glucagon was correlated with the four miRNAs that were downregulated from the induction of hypoglycemia to 4 h later (miR-191-5p, miR-143-3p, let-7b-5p, and let-7g-5p). This revealed a negative correlation of miR-191-5p (r^2^ = 0.25 and *p* = 0.03) and let-7g-5p (r^2^ = 0.22 and *p* = 0.03) in the controls at 4h post-hypoglycemia. In T2D, miR-143-3p correlated negatively with glucagon at baseline (r^2^ = 0.22 and *p* = 0.04) and upon the hypoglycemia insult (r^2^ = 0.24 and *p* = 0.04). There was no correlation of any of these miRNAs with glucagon at 24 h.

### 2.9. Ingenuity Pathway Analysis

**Figure 1:**
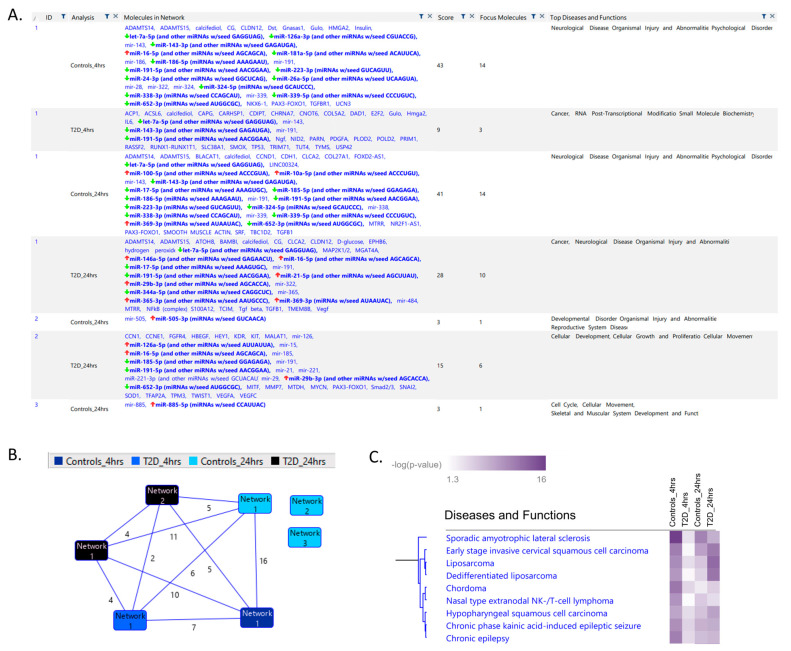

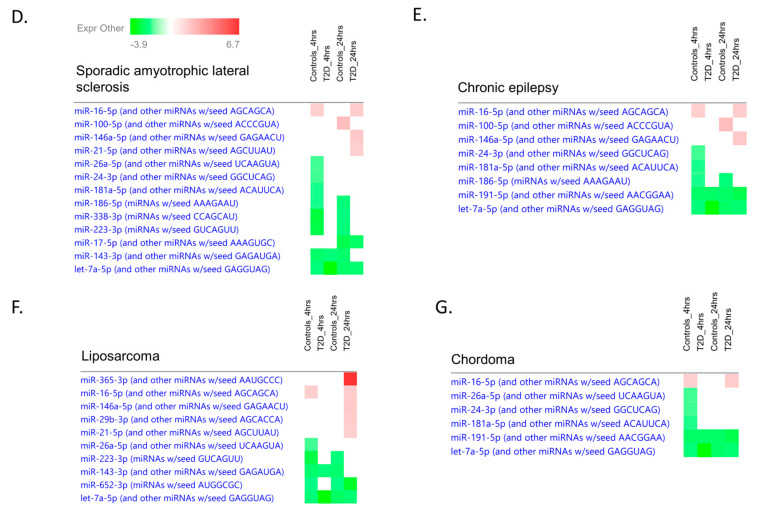
Ingenuity Pathway Analysis (IPA) of significant networks identified. (**A**) List of all the significant networks for the control and type 2 diabetes (T2D) groups at 4 h and 24 h post-hypoglycemia is shown in the “Analysis” column. The “Molecules in Network” column shows the miRNAs present in the data in bold with the expression direction shown with arrows. Other listed genes interact directly or indirectly with the miRNAs in the dataset. The “Score” column is the negative exponent of the right-tailed Fisher’s Exact Test. The “Focus Molecules” column displays the number of miRNAs in our data in the network. The “Top Diseases and Functions” shows the diseases linked to the genes in each network. (**B**) The number of common genes between networks in the different groups is shown by the number at the line midpoint connecting two nodes. Networks without lines to other networks have no shared genes. (**C**) Clustering of the top diseases and functions linked to the significant miRNAs across the different conditions. The *p*-value is that of the association between the disease and the miRNA as calculated by IPA software. (**D**–**G**) Each heatmap shows the expression of the miRNAs in the indicated disease condition across the different conditions.

An Ingenuity Pathway Analysis (IPA) was conducted on the topmost altered miRNAs in the subjects at 4 h and 24 h post-hypoglycemia. Several significant networks were identified between significantly altered miRNAs and other genes/molecules that are linked to diseases and functions in the different conditions (Figure 1A). A *p*-value score for any given network indicates the probability that the incorporated molecules are present in the network by random chance. In T2D at 4 h, neurological disease and organismal injury and abnormalities were highlighted (score *p* < 0.00002). Interestingly, in T2D at 24 h, two distinct pathways were evident in the T2D subjects. The first pathway was neurological disease and organismal injury and abnormalities (score *p* < 1.2^−10^), and the second was cellular development, cellular growth and proliferation, and cellular movement (score *p* < 0.000005). Interestingly, at both the 4 h and 24 h time points, the controls had significantly more molecules and higher significant networks involved in “Diseases and Functions” (Figure 1A), suggesting a disrupted response to hypoglycemia. Note that these pathways are not necessarily independent and genes could be shared between them, as shown in Figure 1B.

**Figure 2:**
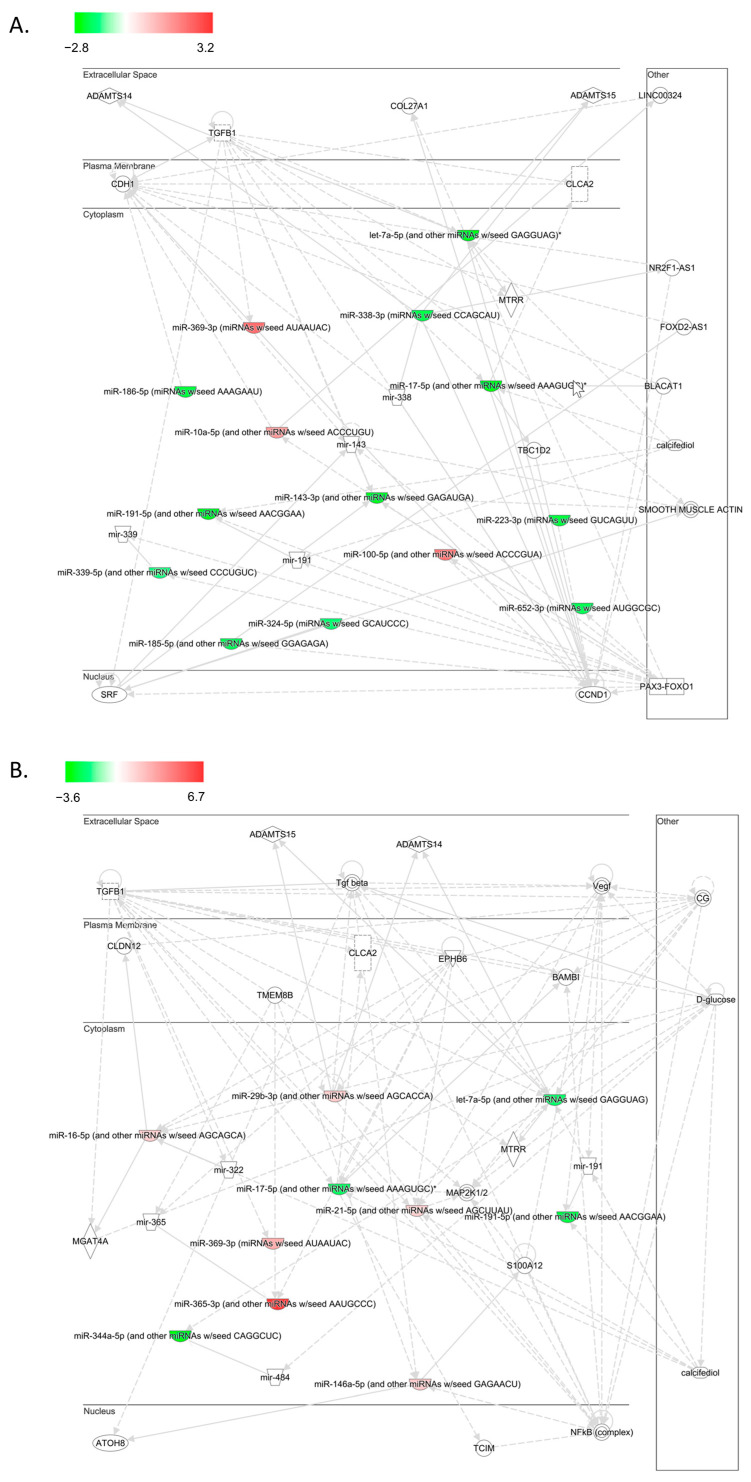
Ingenuity Pathway Analysis (IPA) of the most significant network (network 1) 24 h post-hypoglycemia in controls (**A**) and type 2 diabetes (T2D) (**B**). The networks are depicted in terms of cellular localization. Colored nodes show the expression level of the miRNA. White nodes are those that were not measured in our data or were non-significant. Interactions between nodes (edges) are either direct (solid lines) or indirect (dashed lines).

Furthermore, using the IPA software, we can also examine more specific networks. Figure 1C shows the significance of specific networks across the different conditions for the top network cluster. These specific networks are involved in both cancer and neurological disease. The expression of specific miRNAs in two neurological pathways (Figure 1D,E) and two cancer pathways (Figure 1F,G) is also shown. The specific relationship between the altered miRNAs and other genes/molecules can be visualized with pathway diagrams as shown in Figure 2A for the controls at 24 h and in Figure 2B for T2D at 24 h. These correspond to network 1 in Figure 1A for each condition. These pathway diagrams show a possible complex interplay between altered miRNAs and transcription factors, cytosolic and plasma membrane genes, and extracellular factors.

## 3. Discussion

This study extends the work already published from the same clinical study and performed in the same patient population that reported changes in miRNAs from baseline to the point of hypoglycemia [23] and, further, extends the work of others [24]. Our hypothesis that hypoglycemia would induce miRNA was demonstrated with the primary outcome. Moreover, the novelty of this current study is that it reports on the post-hypoglycemia follow-up period to 24 h post-hypoglycemia and demonstrates that miRNAs are both up-regulated and down-regulated at 4 h following a hypoglycemic episode and that changes in the miRNAs are still evident after 24 h. The correlation of three of the altered miRNAs (miR-191-5p, miR-143-3p, and let-7g-5p) revealed an association with the counter-regulatory response and each of these miRNAs is known to be associated with energy homeostasis. A previous small hypoglycemia study restricted to 14 patients with type 2 diabetes alone using selective miRNA analysis based on platelet expression revealed that an upregulation of the miRNA may be seen at 1 day and 7 days, though an earlier time course miRNA analysis within the first 24 h period was not undertaken [24]; unfortunately, it is not possible to directly compare our study with this study as the degree and duration of hypoglycemia differed between studies. Hypoglycemia in normal control subjects is an unphysiological event but, as was previously reported, the miRNA changes were pronounced at the point of hypoglycemia [23], with more miRNA altered in the controls (nine miRNAs) than in type 2 diabetes (no miRNA changes). This suggests that the physiological response in the T2D subjects in this early phase had been blunted and delayed despite the relatively short duration of disease, which is a feature reported to correspond to a long duration of disease for other physiological parameters [25]. No changes in the miRNAs that passed FDR occurred in the relatively acute reduction in glucose from baseline to hypoglycemia (which was over a period of approximately one hour) for either the controls or T2D patients. Here, miRNA changes were first measured at 4 h post-hypoglycemia; therefore, the miRNA changes did occur within that time period, but the exact timing for each individual miRNA is not known. However, this does indicate that the miRNA response can occur within the relatively short time frame of four hours following a hypoglycemic insult.

Within the time frame of hypoglycemia to 4 h after its induction, there were four miRNAs that were common to both the control and T2D cohorts (hsa-miR-191-5p, hsa-let-7b-5p, miR-let-7g-5p, and hsa-miR-143-3p), which likely indicates the commonality of the response to hypoglycemia. Here, we focus primarily on the acute hypoglycemic episode up to the 4 h post-hypoglycemia timepoint: whilst many more miRNAs were altered in the controls from the induction of hypoglycemia to 4 h later, the four miRNAs that were common to the controls and T2D were a downregulation in miR-191-5p, miR-let-7b-5p, miR-143-3p, and miR-let-7g-5p. All four of these miRNAs have been associated with energy homeostasis. MiRNAs of the let-7 family participate in energy regulation and glucose metabolism [26,27], with the restoration of miR-7a-5b levels resulting in almost complete restoration of the sympathoadrenal response [28]. The upregulation of miR-143 is seen in the livers of obesity mouse models [27]. MiR-191 has also been shown to be highly expressed in the hypothalamus [27] and is connected to early changes in glycemic homeostasis [29]. Therefore, the notion that miRNA’s ability to affect energy metabolism may reverse and protect against hypoglycemia over a prolonged period is not surprising.

As a secondary outcome measure, elements of the counter-regulatory response were investigated. Glucagon was correlated with three miRNAs that were downregulated from the induction of hypoglycemia to 4 h, suggesting that a prolonged glucagon response following hypoglycemia may be potentiated by these miRNAs; however, the correlation with glucagon was lost at 24 h, when glucagon levels had returned to normal, despite the miRNA levels still being different to the baseline.

MiRNAs have been suggested to be important in the epigenetic regulation of critical metabolic, inflammatory, and anti-angiogenic pathways in T2D that may contribute to the development of complications [30]. Here, we show that serum miR-191-5p decreased equally in the controls and T2D at 4 h and 24 h; in non-hypoglycemic conditions, serum miR-191-5p has been shown to be decreased in T2D compared to the controls and has been reported to be a potential marker of cardiovascular disease in the T2D population [31] through its effects on platelet function and reactivity [32]. MiR-191 has been reported to modulate angiogenesis and cellular migration through the paracrine regulation of zonula occludens-1, thereby delaying the tissue repair process in T2D [33]. MiR-191-5p and another miRNA that was downregulated in this study, namely, miR-let-7g-5p, were previously shown to be downregulated in hypertensive patients with metabolic syndrome and serve as independent predictors of chronic kidney disease through an association with large vessel stiffening and the estimated glomerular filtration rate [34]. Here, miR-191 was decreased at 4 h and this persisted at 24 h, suggesting that its effect on platelet function, and hence increased cardiovascular risk, would continue for at least a 24 h period.

MiR-143 was downregulated in this study in response to hypoglycemia. MiR-143 has a particular function in controlling and regulating the metabolism of energy and lipids and its increase is associated with the development of insulin resistance through proinflammatory signaling pathways, whilst its inhibition protects against insulin resistance [35].

MiR-let-7b has been shown to have an inhibitory role in inflammatory cytokine (TNF and IL-6) expression in monocytes and it was reported that let-7b exhibited differing expression patterns in inflamed versus healthy tissues [36].

Others have shown that miR-7b downregulates neutrophil TLR4, provoking a decrease in the proinflammatory cytokines IL-6, IL-8, and TNF-alpha, and an upregulation of anti-inflammatory IL-10 [37]. Therefore, the downregulation seen here with hypoglycemia may be detrimental, potentially resulting in a prolonged increase in inflammatory cytokines.

There were changes in the number of miRNAs expressed from baseline to 24 h compared to the period from acute hypoglycemia to 24 h, but there were seven miRNAs common to both timeframes and cohorts. Here, we will focus primarily on the acute hypoglycemic episode to the 24 h post-hypoglycemia timepoint: whilst there were a similar number of miRNAs altered in the controls and T2D from the induction of hypoglycemia to 24 h, the six altered miRNAs that were common to the controls and T2D were a downregulation in miR-93-5p, miR-let-7b-5p, miR-191-5p, miR-185-5p, and miR-652-3p, and an upregulation in miR-369-3p. It is of interest that miR-191-5p, miR-143-3p, and miR-let-7b-5p that were seen to be downregulated at 4 h continued to be downregulated at 24 h, whilst miR-let-7g-5p returned to baseline in T2D at 24 h but remained low in the controls. As noted above, these three miRNAs have been associated with energy homeostasis and this may suggest that their effects towards promoting a positive energy balance to address hypoglycemia last longer than expected, and it is well recognized that hunger is one of the symptoms of hypoglycemia.

For those miRNAs where there were persistent changes at baseline compared to control at 24 h, we do not know how long those changes persisted nor, therefore, the duration of the potential metabolic or cardiovascular risk sequelae following the hypoglycemic insult; however, in a previous hypoglycemic study, these changes persisted up to seven days [24].

As noted above, each miRNA may cause multiple gene inhibition and many of the significant changes seen in their levels in this study highlight their downregulation, which may translate into enhanced protein translation. Upregulation was seen in several miRNAs, though only miR-369-3p was common to both the controls and T2D and upregulated at 24 h, suggesting that the regulation of those protein pathways for this miRNA was inhibitory. MiR-369 is also found in the hypothalamus arcuate nucleus that is involved in energy homeostasis [38], which is in accordance with the miRNAs and their potential functions as noted above [38].

The IPA revealed no substantially apparent differences between the controls and T2D at 4 and 24 h among the most significant pathways, with most pathways being shared by both cohorts. Whilst cancer-related pathways were found to be linked to the significant miRNAs in both groups and at all timepoints, this is likely a reflection of the fact that most miRNA work has had a cancer-related bias and is likely not relevant to glucose control per se. At 4 h, miRNA changes encompassed neurological disease and organismal injury and abnormalities and the association of miRNA changes with this pathway became more pronounced and significant at 24 h. It is interesting that at 24 h, two distinct pathways were evident in the T2D subjects: neurological disease, which includes Alzheimer’s disease (which is well-known to associate with type 2 diabetes) [39], and organismal injury and abnormalities, which is firstly associated with diabetes complications, while cellular development, cellular growth and proliferation, and cellular movement represent the second association. The increased association of these conditions at 24 h is indicative that there is ongoing pathway disruption at 24 h, and likely beyond, following a single hypoglycemic episode. This may suggest that multiple hypoglycemic episodes within a relatively short timeframe may compound and aggravate these effects, leading to clinical sequelae such as a cardiovascular event.

Kallistatin has been reported to inhibit oxidative stress-induced cellular senescence by upregulating miR-let-7g synthesis in signaling pathways in human endothelial cells via the eNOS pathway [40]. Given that oxidative stress is suggested to be a crucial mechanism underlying the development of diabetes-related complications, in accordance with miR-let-7b, the downregulation seen here in miR-let-7g with hypoglycemia may be detrimental, with the potential to provoke a prolonged increase in oxidative stress as a consequence.

The determination of the common miRs that were altered at 24 h showed that miR-93-5p, miR-185-5p, and miR-652-3p were decreased whilst miR-369-3p was increased.

It has been shown that miR-93-5p overexpression is associated with enhanced insulin resistance and that it may modulate the inflammatory response in diabetic retinopathy [41]. The expression of miR-93-5p was shown to be decreased in diabetic nephropathy, again under the regulation of lncRNA, and its inhibition reduced fibrosis and inflammation with a reduction in TNF-α, IL-6, and IL-1β [42]. In accordance with miR-let-7b and miR-let-7g, the downregulation of miR-93 seen here with hypoglycemia may, therefore, again be detrimental, with the potential for a prolonged increase in proinflammatory cytokines.

In vitro studies have implicated miR-185-5p in the development of diabetes nephropathy via its influence on inflammatory responses and fibrosis pathways [43]. Higher levels of miR-185 have been shown in the vitreous samples of patients with proliferative diabetic retinopathy [44]. The inhibition of miR-185 by hypoglycemia may then impact upon the development of diabetes retinopathy, particularly if hypoglycemic episodes are recurrent, constituting a negative consequence that sometimes results from the tight optimization of diabetes control.

Circulating miR-162 has been shown to be reduced in T2D, though it was not found to be lower in this study at the baseline. It has been reported that miR-162 is regulated by cytokines [45]; however, its relationship to metabolic dysfunction and diabetes-related complications is unclear.

The upregulation of miR-369-3p has been shown to decrease inducible nitric oxide synthase and the release of TNFα, IL-6, IL-12, IL-1α, and IL-1β in response to the stress of lipopolysaccharide in vitro, whilst it increased the production of anti-inflammatory cytokines such as IL-10 and IL-1RA [46]. Whilst the relationship of miR-369 to metabolic dysfunction and diabetes-related complications is unclear, its increase seen here following hypoglycemia may suggest a protective role to offset the potential deleterious effects of the miRNAs noted above that are modulated at the same time.

The data showed glucagon elevation in response to hypoglycemia in both the control and T2D cohorts, as would be expected in the counter-regulatory response. Cortisol and growth hormone responses are well-recognized as occurring following hypoglycemia’s onset, though these were not measured in this study. In a prior study in a separate patient cohort, we evaluated nor-metadrenaline and metadrenaline responses to hypoglycemia and reported differences at 24 h [9]; therefore, these were not measured in this study.

The T2D patients enrolled here were relatively treatment naïve and possessed a short disease duration, which represents a strength of this study. Its limitations include the modest study numbers, as a larger cohort may have revealed further miRNA changes over the time course; however, this study was undertaken as a pilot, as there were no previous studies on which to base a power analysis for a definitive study. Additionally, the time course was restricted to the baseline, the hypoglycemic event, and 4 and 24 h post-hypoglycemia; however, such a severe hypoglycemic event as was induced here would be expected to provoke changes in miRNA levels that would be apparent during that timeframe. Despite the T2D subjects being more obese, it is highly likely that this did not affect the changes of these miRNAs in response to hypoglycemia. HbA1c, total cholesterol, triglycerides, and HDL-cholesterol differed between groups, but it is unlikely that those differences would alter the response to hypoglycemia [47], though this would need confirmation. As the study cohort was entirely Caucasian, it may not be possible to generalize the results to patients of other ethnicities. Considering the alterations in miRNAs in the control cohort at 24 h, it would be interesting in future studies to lengthen this follow up period to ascertain when the alterations normalize back to the baseline, as well as the temporal changes and interplay with counter-regulatory mechanisms, oxidative stress, and proinflammatory pathways. It should also be noted that the circulating levels of miRNAs may not be reflective of their levels or activity at the cellular level. Determining miRNA changes at the tissue level would be crucial to elucidate the significance of their alteration in the circulation and their application in the clinical arena and should be undertaken in the future to address this. However, the pronounced changes seen in the miRNA at 4 h and 24 h may allow for a panel of miRNAs to be produced that can be clinically applied to identify a hypoglycemic event in the face of diagnostic uncertainty.

## 4. Methods

### 4.1. Study Design

This parallel prospective study was undertaken on 46 adult subjects, equally divided into T2D and controls (n = 23 for each cohort), at the Hull Royal Infirmary Diabetes Centre; studies were undertaken from March 2017 to January 2018. As previously described [48], “Age matched subjects were recruited; all subjects were Caucasian, aged 40–70 years. The duration of diabetes was <10 years and all T2D subjects were on a stable dose of medication (metformin, statin and/or angiotensin converting enzyme inhibitor/angiotensin receptor blocker) over the prior 3-month period. For T2D patient inclusion, only metformin as anti-diabetic therapy was allowed; other inclusion criteria were HbA1c < 10% (86 mmol/mol), with no hypoglycemic unawareness or hypoglycemia within the prior 3-month period, and no diabetes related complications. In the control group, diabetes was excluded with an oral glucose tolerance test. All subjects had a body mass index (BMI) between 18 and 49 kg/m^2^, normal renal and hepatic biochemical indices and no prior history of cancer, nor any contraindication to insulin infusion to achieve hypoglycemia (ischemic heart disease, epilepsy, seizure history, drop attacks, history of adrenal insufficiency and treated hypothyroidism)”.

### 4.2. Study Participants

Medical history, clinical examination, routine blood tests, and an electrocardiogram was performed for all participants. Hypoglycemia was induced by a continuous insulin infusion as previously detailed [5] with blood samples taken upon induction of hypo (time 0), 4 h, and 24 h post-hypoglycemia. After 4 h, participants were provided lunch and the T2D cohort was given their morning diabetes medications. Evening medication was taken by patients as per prescription. At 24 h following the induction of hypoglycemia, patients withheld their medications until they completed the blood tests in the fasted state. Then, breakfast was provided, and, prior to discharge, blood glucose was checked using a glucose analyzer (HemoCue glucose 201+) to ensure normal levels, together with other vital signs.

All participants provided written informed consent. The trial was approved by the North West-Greater Manchester East Research Ethics Committee (REC number:16/NW/0518), registered at www.clinicaltrials.gov (NCT03102801) on 6 April 2017, and was “conducted according to the Declaration of Helsinki”.

### 4.3. Insulin Infusion

The insulin infusion was performed as previously detailed [5]. “Following an overnight fast, 30–60 min prior to the commencement of the test (08:30 h), bilateral ante-cubital fossa indwelling cannulas were inserted. To induce hypoglycemia, soluble intravenous insulin (Humulin S, Lilly, UK) was given in a pump starting at a dose of 2.5 mU/kg body weight/min with an increment of 2.5 mU/kg body weight/min every 15 min by hypoglycemic clamp, until two readings of capillary blood glucose measured by a glucose analyser (HemoCue glucose 201+)  ≤2.2 mmol/L (<40 mg/dL) or a reading of ≤2.0 mmol/L (36 mg/dL) was achieved. Initially, patients with T2D were clamped to euglycemia (5 mmol/L; 90 mg/dL), then subsequently to hypoglycemia. The blood sample schedule was timed subsequently in respect to the time point that hypoglycemia occurred. Following the identification of hypoglycemia, intravenous glucose was given in the form of 150 mL of 10% dextrose and a repeat blood glucose check was performed after 5 min if blood glucose was still <4.0 mmol/L”.

### 4.4. Biochemical Markers

As previously described [48], “blood samples were separated immediately by centrifugation at 2000 *g* for 15 min at 4 °C, and the aliquots were stored at −80 °C, within 30-min of blood collection, until batch analysis. Fasting plasma glucose (FPG), total cholesterol, triglycerides, and high-density lipoprotein (HDL) cholesterol levels were measured enzymatically using a Beckman AU 5800 analyser (Beckman-Coulter, High Wycombe, UK)”.

### 4.5. Proteomic Analysis

As detailed previously [48], “Slow Off-rate Modified Aptamer (SOMA)-scan proteomics was undertaken for protein measurement as previously detailed [6] and proteins quantified by hybridization to custom arrays of SOMAmer-complementary oligonucleotides. Normalization of raw intensities, hybridization, median signal and calibration signal were performed based on the standard samples included on each plate, as previously described [6]. Post-hypoglycemia timepoints were at 4 and 24 h”.

### 4.6. RNA Extraction from Human Plasma

As described previously [23], “Total RNA was extracted using the MagMAX™ *mir*Vana™ RNA Isolation Kit (Thermo Fischer Scientific, Waltham, MA, USA) on an automated KingFisher instrument (Thermo Fischer Scientific, Waltham, MA, USA). The MagMAX™ *mir*Vana™ RNA isolation kit is a magnetic bead-based kit that uses MagMAX magnetic-bead technology for efficient isolation of total RNA from plasma samples from 100 µL of human plasma”.

### 4.7. Expression Analysis of microRNA by TaqMan OpenArray Human Advanced MicroRNA Panel

A total of 2 µL of the extracted total RNA was used for cDNA conversion using TaqMan™ Advanced miRNA cDNA Synthesis Kit (Thermo Fischer Scientific, Waltham, MA, USA) as per manufacturer’s recommended protocol. Given the low abundance of miRNA in plasma samples, cDNA was pre-amplified prior to the final real-time PCR step. The 1:20 diluted cDNA was mixed with 2X TaqMan OpenArray Real-Time Master Mix solution to perform real-time PCR on the OpenArray plate. A 5 µL sample of PCR reactions was distributed into each well of the 384-well plate and samples with the master mix were loaded from the 384-well sample plate onto the OpenArray plate using the OpenArrayAccuFill System. Finally, the PCR was run on the QuantStudio 12K Flex Real-Time PCR System (Thermo Fischer Scientific, Waltham, MA, USA). The miRNA measurements were conducted in two phases: (1) profiling and (2) validation. Initially, profiling was conducted using pooled samples (4 in each group) obtained from 12 subjects in both control and diabetes groups at baseline and upon hypoglycemia. Profiling for miRNA was performed using The TaqMan^®^ OpenArray^®^ Human MicroRNA Panel which is a fixed-content panel that contains 754 well-characterized human miRNA sequences. The samples were loaded on to the chip using the automated Accufill^TM^ System (Thermo Fischer Scientific, Waltham, MA, USA) and were run on QuantStudio™ 12K Flex Real-Time PCR System for determining the miRNA expression. Profiling experiments were conducted in a set of pooled samples taken from all timepoints and using default miRNA panels that consisted of 754 miRNAs. Based on the results from these profiling experiments, the most commonly expressed miRNAs (equaling 96 miRNAs) found in the majority of the pooled samples in both groups were selected for the validation experiment. Validation was conducted in all the individual samples from each group, using custom-designed miRNA panels. The miRNA expressions were quantified using the ExpressionSuite Software (Thermo Fischer Scientific, Waltham, MA, USA) data-analysis tool that utilizes a comparative Cτ (ΔΔCτ) method to accurately quantify relative miRNA expressions. “ExpressionSuite generates the results in the form of Rq values, which are equivalent to the log2 scale; therefore, any changes indicate a log2 fold change”.

### 4.8. Ingenuity Pathway Analysis

Ingenuity Pathway Analysis (IPA) software (Qiagen, Germantown, MD, USA) enables data analysis plus integration of data procured from an assemblage of experimentally derived datasets, such as gene expression and miRNA. Here, IPA (content version 73620684, Release Date: 12 March 2022) was performed to display the pathways linked to the top altered miRNAs in T2D and control subjects as featured in this study.

### 4.9. Statistics

As detailed previously [23], “There was no information regarding changes in miRNA on which to base a sample size calculation. For such pilot studies, Birkett and Day [49] suggest a minimum of 20 degrees of freedom to estimate variance from which a larger trial could be powered, hence 23 subjects in each group were recruited. Statistical analysis was performed using SPSS (v22, Chicago, IL, USA). Descriptive data is presented as mean ± SD for continuous data and n (%) for categorical data. *t*-tests or Mann Whitney tests were used to compare means/medians where appropriate. Linear associations were assessed using Pearson’s correlation test. Mean normalization was performed before analysis using the global mean of each miRNA. Using GenEx software version 5.3 that accompanied the Bioanalyzer, normalization was performed to achieve a global mean of all miRNAs with Ct below 32. An unpaired *t*-test was used to test the changes between T2D and controls in miRNA levels. False discovery rates (FDR) of q < 0.05 were considered significant”.

## 5. Conclusions

In conclusion, this study demonstrates, for the first time, that hypoglycemia affected miRNA levels at 4 h post-hypoglycemia that reflected metabolic/coagulation pathways and that differed in the controls at 4 h post-hypoglycemia versus T2D. However, a similar number of miRNAs were altered at 24 h in each cohort, suggesting both a potential delay in the pathophysiological hypoglycemic response in T2D and that miRNAs may potentiate and prolong the physiological response following a hypoglycemic event for over 24 h. Furthermore, many of the miRNAs that were altered in response to hypoglycemia have been shown to modulate inflammatory and oxidative stress pathways, whose prolonged activation is detrimental.

## Figures and Tables

**Table 1:** Demographic and clinical characteristics of the study participants [23].

Baseline	Type 2 Diabetes (n = 23)	Controls (n = 23)	*p*-Value
Age (years)	64 ± 8 (66)	60 ± 10 (63)	0.15
Sex (M/F)	12/11	11/12	0.77
BMI (kg/m^2^)	32 ± 4 (32)	28 ± 3(27)	0.001
Duration of diabetes (years)	4.5 ± 2.2 (5.0)	N/A	
HbA1c (mmol/mol)	51.2 ± 11.4 (50.0)	37.2 ± 2.2 (37.0)	<0.0001
HbA1c (%)	6.8 ± 1.0 (6.7)	5.6 ± 0.2 (5.5)	<0.0001
Total cholesterol (mmol/L)	4.2 ± 1.0 (4.1)	4.8 ± 0.67 (4.9)	0.02
Triglyceride (mmol/L)	1.7 ± 0.7 (1.5)	1.34 ± 0.6 (1.3)	0.06
HDL-cholesterol (mmol/L)	1.1 ± 0.3 (1.1)	1.5 ± 0.4 (1.4)	0.002
LDL-cholesterol (mmol/L)	2.27 ± 0.8 (2.1)	2.7 ± 0.7 (2.8)	0.06
CRP (mg/L)	3.0 ± 2.7 (1.9)	5.1 ± 10.3 (2.1)	0.33

BMI: Body mass index, BP: Blood pressure, HDL-cholesterol: High-density lipoprotein cholesterol, LDL-cholesterol: Low-density lipoprotein cholesterol, CRP: C-reactive protein, and HbA1c: Hemoglobin A1c. Data shown as mean ± SD (median).

## Data Availability

All the data for this study will be made available upon reasonable request to the corresponding author.

## Supplementary Materials

The following supporting information can be downloaded at: https://www.mdpi.com/article/10.3390/ijms232314696/s1.
