# Supplementary material for: MicroRNA Changes Up to 24 h following Induced Hypoglycemia in Type 2 Diabetes
Source: Int J Mol Sci. 2022 Nov 24;23(23):14696. doi: 10.3390/ijms232314696 (PMC9736413; doi:10.3390/ijms232314696)
Supplement: Supplementary file 1 [file ijms-23-14696-s001.zip › ijms-2010621-supplementary.pdf]

Figure S1

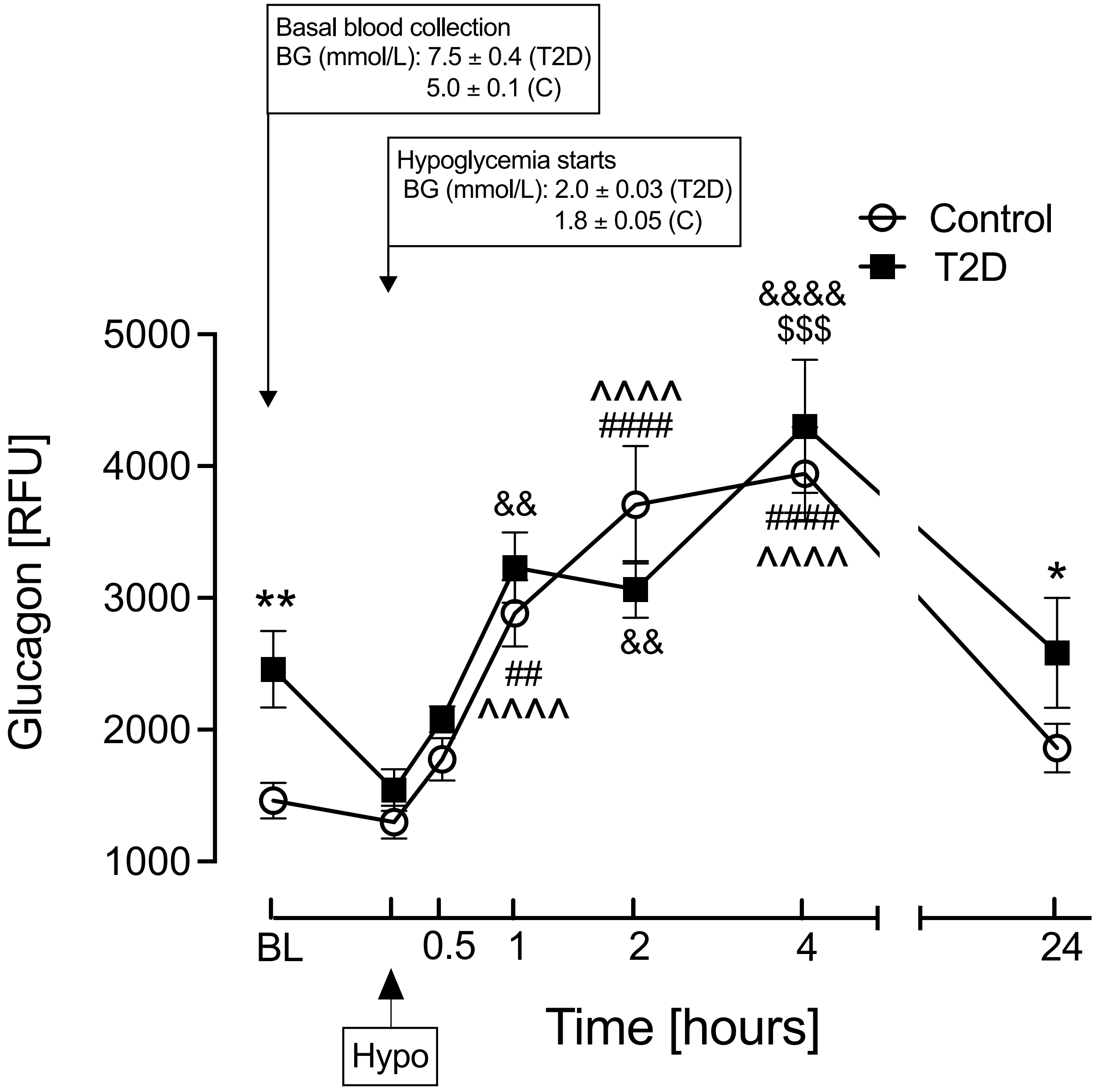

Basal blood collection
BG (mmol/L): 7.5 ± 0.4 (T2D)
5.0 ± 0.1 (C)
Hypoglycemia starts
BG (mmol/L): 2.0 ± 0.03 (T2D)
1.8 ± 0.05 (C)
Control
T2D
Glucagon [RFU]
5000
4000
3000
2000
1000
**
&&
##
^^^^
^^^^
####
&&
&&&&
$$$
####
^^^^
*
BL
0.5
1
2
4
24
Hypo
Time [hours]

Figure S1. Blood levels of glucagon at baseline, after induction of hypoglycemia and in the post-hypoglycemia follow-up period in control and Type 2 Diabetes (T2D) subjects. Blood sampling was performed at Baseline (BL), at hypoglycemia (0 min) and at post-hypoglycemia timepoints (0.5-hours, 1-hour, 2-hours, 4-hours and 24-hours) for controls (white circles) and T2D (black squares). Somalogic proteomic analysis was undertaken to detect the levels of plasma proteins.

Statistics: *, p< 0.05, **, p< 0.01, T2D vs control.

Changes in protein levels within group (control and T2D) relative to baseline and hypoglycemia are also shown. Statistics: control, baseline to hypoglycemia and post-hypo timepoints: ^^^^ p<0.0001; Control, hypoglycemia to subsequent timepoints: ## p<0.01, ### p<0.001, #### p<0.0001. T2D, baseline to hypoglycemia and post-hypo subsequent timepoints: && p<0.01, &&&& p<0.0001; T2D, hypoglycemia to post-hypo subsequent timepoints: $$$ p<0.001.

RFU-relative fluorescent units; BG-blood glucose; Hypo-hypoglycemia

Table S1. MiRNAs that changed from baseline to 4-hours post-hypoglycemia (A) and from baseline to 24-hours post-hypoglycemia in control (n=23) and type 2 diabetes (T2D, n=23) subjects in the validation experiment.

A. It can be seen that the changes in miRNA from baseline to 4-hours post-hypoglycemia closely reflect the changes from hypoglycemia to 4-hours post-hypoglycemia, as is shown in Table 2, with more miRNA changes (n=13) seen in control subjects versus subjects with T2D (n=4). All miRNAs were downregulated in controls while, in T2D, 3 miRNAs were downregulated and only 1 was upregulated. Significant changes in miRNAs are shown by fold change (Rq).
Those miRNAs highlighted in blue were common to both control and T2D. In accord with the changes in miRNA from the point of hypoglycemia to 4-hours post-hypoglycemia (Table 2), 3 of the 4 miRNAs altered in T2D from baseline to 4-hours post-hypoglycemia were also altered in controls, and all were downregulated. FDR, false discovery rate; Rq, relative level of miRNA expression.

B. The number of miRNAs that were altered from baseline to 24-hours post-hypoglycemia was similar in control (n=16) and T2D (n=15) subjects. In controls, the majority of miRNAs were downregulated (n=12) with only 4 upregulated. In T2D, half were downregulated (n=8) and half upregulated (n=7). Those miRNAs highlighted in blue were common to both control and T2D. Significant changes in miRNAs are shown by fold change (Rq). FDR, false discovery rate; Rq, relative level of miRNA expression.

A

**Control Subjects (n=23)**				**T2D subjects (n=23)**			
**Baseline to 4 hours**			n=13	**Baseline to 4 hours**			n=4
**Target Name**	**Rq (fold change)**	**FDR**	**Regulation**	**Target Name**	**Rq (fold change)**	**FDR**	**Regulation**
hsa-miR-191-5p_477952_mir	2.8	0	Down	hsa-miR-191-5p_477952_mir	2.2	0.001	Down
hsa-let-7b-5p_478576_mir	2.8	0	Down	hsa-let-7b-5p_478576_mir	3.7	0.002	Down
hsa-miR-652-3p_478189_mir	2.7	0.001	Down	hsa-miR-143-3p_477912_mir	2.3	0.004	Down
hsa-miR-223-3p_477983_mir	2.6	0.006	Down	hsa-miR-365a-3p_478065_mir	7.8	0.029	Up
hsa-miR-338-3p_478037_mir	2.4	0.026	Down				
hsa-miR-324-5p_478024_mir	2.4	0.003	Down				
hsa-miR-186-5p_477940_mir	2.2	0	Down				

hsa-miR-17-5p_478447_mir	2.1	0.029	Down
hsa-miR-143-3p_477912_mir	2.0	0.018	Down
hsa-let-7g-5p_478580_mir	1.8	0.002	Down
hsa-miR-26a-5p_477995_mir	1.8	0.002	Down
hsa-miR-151a-3p_477919_mir	1.8	0.018	Down
hsa-miR-339-5p_478040_mir	1.7	0.023	Down

B

**Control Subjects (n=23)**				**T2D subjects (n=23)**			
**Baseline to 24 hours**			n=16	**Baseline to 24 hours**			n=15
**Target Name**	**Rq (fold change)**	**FDR**	**Regulation**	**Target Name**	**Rq (fold change)**	**FDR**	**Regulation**
hsa-miR-17-5p_478447_mir	3.4	0	Down	hsa-miR-484_478308_mir	3.4	0.035	Down
hsa-let-7b-5p_478576_mir	3.1	0	Down	hsa-miR-652-3p_478189_mir	3.2	0.008	Down
hsa-miR-93-5p_478210_mir	2.8	0.001	Down	hsa-miR-93-5p_478210_mir	2.9	0.012	Down
hsa-miR-191-5p_477952_mir	2.7	0	Down	hsa-miR-191-5p_477952_mir	2.5	0.01	Down
hsa-miR-652-3p_478189_mir	2.6	0	Down	hsa-miR-106b-5p_478412_mir	2.3	0.014	Down
hsa-miR-186-5p_477940_mir	2.5	0	Down	hsa-let-7b-5p_478576_mir	2.2	0.01	Down
hsa-miR-20a-5p_478586_mir	2.5	0.001	Down	hsa-miR-185-5p_477939_mir	2.0	0.007	Down
hsa-miR-185-5p_477939_mir	2.2	0.019	Down	hsa-miR-143-3p_477912_mir	1.6	0.034	Down
hsa-let-7g-5p_478580_mir	2.0	0.008	Down	hsa-miR-424-5p_478092_mir	1.7	0.016	Up
hsa-miR-143-3p_477912_mir	2.0	0.002	Down	hsa-miR-146a-5p_478399_mir	1.8	0.014	Up
hsa-miR-324-5p_478024_mir	1.9	0.005	Down	hsa-miR-126-5p_477888_mir	1.8	0.003	Up
hsa-miR-151a-3p_477919_mir	1.6	0.029	Down	hsa-miR-21-5p_477975_mir	1.9	0	Up
hsa-miR-125b-5p_477885_mir	1.5	0.032	Up	hsa-miR-10a-5p_479241_mir	2.2	0.008	Up
hsa-miR-505-3p_478145_mir	2.3	0	Up	hsa-miR-369-3p_478067_mir	3.6	0.019	Up
hsa-miR-369-3p_478067_mir	2.4	0.021	Up	hsa-miR-365a-3p_478065_mir	13.5	0.007	Up
hsa-miR-885-5p_478207_mir	3.9	0.004	Up				
